# Oxygen Adsorption on Polar and Non-Polar Zn:ZnO Heterostructures from First Principles

**DOI:** 10.3390/ma16031275

**Published:** 2023-02-02

**Authors:** António Castro, Sebastian Calderon, Luís Marques

**Affiliations:** 1 Center of Physics of Minho and Porto Universities, University of Minho, Campus de Gualtar, 4710-057 Braga, Portugal; 2 Laboratory of Physics for Materials and Emergent Technologies, LapMET, University of Minho, 4710-057 Braga, Portugal; 3 INL, International Iberian Nanotechnology Laboratory, Av. Mestre José Veiga, 4715-330 Braga, Portugal

**Keywords:** Zn:ZnO heterostructures, density functional theory, O_2_ adsorption

## Abstract

Zn:ZnO nanostructures have been studied extensively due to their potential use in many applications, such as oxygen scavengers for food packaging applications. Under atmospheric conditions, ZnO grows on the surface of Zn via an oxidation process. The mechanisms governing Zn oxidation are still not fully understood, with classical oxidation models, such as the Cabrera Mott, underestimating the oxide thickness of Zn:ZnO core–shell structures. In this work, Ab initio DFT calculations were performed to assess the adsorption properties of oxygen molecules on Zn:ZnO heterostructures to help elucidate the mechanisms involved in the growth of a ZnO film on a Zn substrate. Results suggest that the charge transfer mechanism from the Zn:ZnO heterostructures to the adsorbed oxygen layer can be promoted by two different processes: the electronic doping of ZnO due to the formation of the Zn:ZnO interface and the excess surface charge due to the presence of dangling bonds on the as cleaved ZnO.

## 1. Introduction

ZnO is an important wide band-gap semiconductor that finds applications in different areas, for instance, in solar cells, gas sensors, field emission displays, and UV lasers, among others [1]. ZnO nanoparticles also have raised interest in food packaging applications due to their interesting optical, electrical, and chemical properties, as well as antibacterial and antifungal activity [2]. ZnO films form naturally on metallic zinc surfaces under normal atmospheric conditions. Both ZnO and Zn crystallize on a hexagonal structure; as such ZnO tends to grow epitaxially on the surface of Zn via the oxidation process [3], spontaneously forming Zn:ZnO metal-semiconductor junctions. Due to its potential applications, the synthesis of Zn:ZnO core–shell structures on the micro and nanoscale has been a topic of various studies. Various novel morphologies such as core–shell microspheres [4], hollow microspheres [5], hexagonal nanodisks [6], nanocables [7], nanobelts, and nanotubes [8] have been reported. The oxidation of Zn nanoparticles and consequent formation of Zn:ZnO core–shell structures have been reported, also, due to its potential as an oxygen scavenger for food packaging applications [9]. However, the mechanisms governing Zn oxidation are still not fully understood, with classical oxidation models, such as the Cabrera Mott (CM), underestimating the oxide thickness on Zn:ZnO core–shell structures. In the CM model, it is assumed that electrons the metal substrate transfer across the oxide layer to the adsorbed oxygen species. The accumulation of anionic and cationic species on both sides of the oxide generates an electrical potential, known as the Mott potential (VM), that lowers the migration barriers of ionic species through the oxide [10]. CM theory has been adapted by several authors and applied to describe the growth of the oxide layer in different structures such as nanoparticles [11,12] and nanowires [13]. Experimental results for the ZnO growth on Zn nanoclusters and nanoparticles under ambient conditions were analyzed considering the CM theory [9,14]. While qualitatively the CM theory describes the self-limiting growth kinetics for the nanoparticles under ambient conditions, it tends to underestimate the oxide layer thickness. The selection of the right parameters was pointed out as one of the problems when applying CM based models, mainly the migration barrier (W) and the Mott potential (V_M_). These two parameters are critical to define the oxidation rate, but obtaining a good estimation of their values can be troublesome. Reported values and strategies to obtain parameter estimations from experimental data, can give very different values for W and V_M_ [9]. Several reasons have been pointed out by other authors, referring to limitations due to the phenomena that are included within the CM framework. For instance, the CM model doesn’t consider changes on the electronic properties depending on the particle shape and size, thus affecting the V_M_. In addition, the diffusing species considered might not be correct [14]. Although widely used, the CM model is based on a continuum description of the system and doesn’t consider the atomic configuration nor the electronic structure. In this work, first principles DFT calculations were performed to help elucidate the mechanisms underlying the growth of a ZnO layer on a Zn substrate, focusing on the adsorption of oxygen on top of the polar (0001) and non-polar ($10\bar{1}0$) ZnO surface. The improved knowledge on the oxidation behavior can help to design strategies to control the oxidation process of the metal-semiconductor core–shell structures.

## 2. Materials and Methods

### 2.1. Models

**Figure 1:**
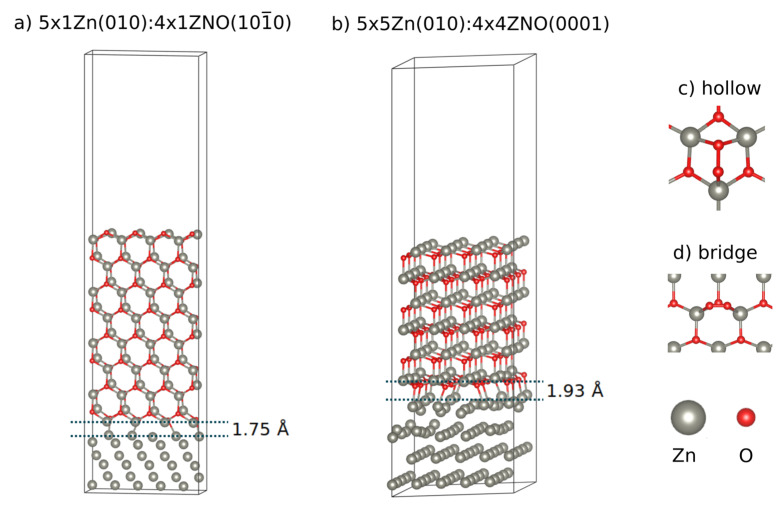
Zn:ZnO supercells used on DFT calculations and high symmetry $O_{2}$ adsorption positions: (**a**) 5×1Zn(010):4×1ZnO($10\bar{1}0$; (**b**) 5×5Zn(001):4×4ZnO(0001); (**c**) hollow adsorption position; (**d**) bridge adsorption position. Gray and red spheres correspond to Zn and O atoms, respectively.

First-principle calculations based on DFT were performed on Zn:ZnO heterostructures to help illustrate the properties of oxygen adsorption on core–shell type nanostructures. Heterostructure models were created using a metallic phase composed of hexagonal close-packed Zn and a semiconductor phase, composed of wurtzite ZnO. Both phases were aligned to the same crystallographic orientation. Two different orientations were selected to create heterostructures containing the 0001 ZnO polar and $10\bar{1}0$ non-polar surfaces. These surfaces belong to the dominant low-index Miller family of hexagonal wurtzite ZnO crystal [15]. Different thicknesses for the ZnO phase were considered to illustrate the initial steps of ZnO growth. Zn presents a closed-packed hexagonal crystal structure with lattice constants a = 0.2665 nm and c = 0.4947 nm, while ZnO exhibits a hexagonal structure with lattice constants a = 0.3249 nm and c = 0.5206 nm. Taking Zn as the reference crystal, the mismatch between the two structures is 21.6% which introduces a large stress on the interface. This mismatch has been shown to be relaxed by misfit dislocations at the interface [16]. As such, 5×5Zn(001):4×4ZnO(0001) and 5×1Zn(010):4×1ZnO($10\bar{1}0$) supercells (Figure 1) were created to reduce the lattice mismatch between the Zn and ZnO phases. The 5×5Zn(001):4×4ZnO(0001) polar ZnO(0001) terminated heterostructures (PZnOTH) were modeled using 4 and 6 bilayers of ZnO(0001) on top of 4 layers of Zn(001), setting the in-plane lattice parameters (ILP) to the same as bulk ZnO. The two bottom layers of Zn(001) were fixed to their bulk positions. The 5×1Zn(010):4×1ZnO($10\bar{1}0$) non polar ZnO($10\bar{1}0$) terminated heterostructures (NPZnOTH), were composed of 4 and 8 bilayers ZnO($10\bar{1}0$) on top of 6 layers of Zn(010), using the bulk ZnO ILP. The four bottom Zn(010) layers were fixed to their bulk positions.

Molecular oxygen adsorption on Zn:ZnO heterostructures was modeled by placing $O_{2}$ molecules atop the ZnO surface, on selected high symmetry adsorption positions, of the created supercells. For the PZnOTH, a monolayer of molecular oxygen with a coverage of ¼ was placed on the hollow position (Figure 1). This is known to be the most stable adsorption position on the ZnO(0001) surface [17]. On the NPZnOTH, a molecular oxygen monolayer with ¼ coverage was placed on the bridge position (Figure 1). The molecular oxygen adsorbs very weakly on non-polar ZnO($10\bar{1}0$) surfaces, however, on doped ZnO, it was shown that the bridge position exhibits the strongest binding energy for the oxygen molecule [18].

### 2.2. DFT Calculations

Calculations with spin polarization were performed within the density functional theory (DFT). The exchange correlation potentials were treated using the Perdew–Burke–Ernzerhof (PBE) parameterization within the general gradient approximation (GGA) [19], as implemented in the Vienna ab initio simulation package (VASP) [20], with the frozen-core projector-augmented-wave (PAW) pseudopotentials [21]. A plane–wave basis set was used with an energy cut-off of 520 eV and Gaussian smearing was used with σ = 0.02 eV. Dispersion forces were taken into account under the DFT-D3 method of Grimme with Becke–Johnson damping [22]. Heterostructure models were built using hcp Zn and wurtzite ZnO optimized unit cells. The calculation details of the structural and electronic properties of Zn and ZnO are shown in the Supplementary Materials, (Table S1, and Figures S1 and S2). Heterostructures were then optimized with the conjugate gradient method, allowing atom positions to relax until atomic forces were less than 0.01 eV/*Å*. The slab dimensions remained unchanged during the relaxation and the Zn bottom layers were frozen to their bulk positions (two layers on the PZnOTH and four on the PZnOTH). Brillouin integration was performed using a Monkhorst–Pack mesh with k-point sampling with a 3 × 11 × 1 mesh for the 5×1Zn(010):4×1ZnO($10\bar{1}0$) and 3 × 3 × 1 for 5×5Zn(001):4×4ZnO(0001). The periodically replicated slabs were separated by a vacuum region of at least 20 Å and a dipole correction was applied perpendicular to the slab surface to avoid interactions between repeated slabs.

The GGA-PBE exchange-correlation of functional tends to severely underestimate the band gap of ZnO and the binding energies of the 3d state of Zn electrons [23]. It has been shown, for several semiconductors, that PBE fails to correctly describe the positions of the valence band maximum (VBM) and the conduction band minimum (CBM). Nevertheless, it can provide reasonable interfacial band offsets due to error cancelation that qualitatively agree with more sophisticated methods and experimental data [24]. To overcome the band gap issue, corrections to the GGA functional (GGA+U), the use of hybrid functionals or GW many body approximation, can be used. However, some aspects must be considered when using these alternative methods. For GGA+U, Hubbard corrections must be applied to Zn 3d and O 2p states in order to obtain a correct band-gap. These corrections have an impact on the oxide structure, causing a decrease in lattice constants [25]. Additionally, since corrections are applied to Zn 3d states, the optimization of the electronic structure of ZnO will also have an impact on metallic zinc properties. The use of Hybrid functionals or metal/semiconductor interfaces can be restricted due to the system size, due to the high computational cost, and often rely on GGA level calculations to obtain optimized structures [24,26]. The same caveats apply to the GW approach, which also limits the spectrum of applications.

Charge densities and electrostatic potentials were analyzed by performing x–y planar-average to obtain a one-dimensional profile on the direction perpendicular to the heterostructure’s surface (z coordinate). Additionally, for the electrostatic potential analysis, the double-macroscopic-average method (or macroscopic potential) [27] was applied to filter potential fluctuations inside the ZnO phase. Atomic structures and volumetric data representations were made using the VESTA software (ver. 3.5.8, Koichi Momma and Fujio Izumi, Tsukuba-shi, Japan) [28]. Bader charges were calculated using the scheme proposed by Henkelman et al. [29] to obtain an approximation of the total electronic charge of an atom.

## 3. Results and Discussion

### 3.1. Zn:ZnO Junctions

Zn:ZnO heterostructures form a metal-semiconductor type of contact. A p-type (or n-type) Schottky Barrier is formed at the metal-semiconductor interface as the result of the difference between the metal Fermi level and the top (bottom) of the semiconductor valence (conduction) band. Depending on the Schottky Barrier height, the junction can form an ohmic or Schottky type of contact [30]. Since the Fermi level of metallic Zinc is located above the CBM of ZnO (forming an ohmic contact), a flow of electrons from the metal to the conduction band of the oxide will occur. Consequently, the Fermi level near the interface will be located above the conduction band resulting in excess electrons, which will drive the system to n-type conditions [31].

The charge transfer occurring at the Zn:ZnO interface can be obtained from the electron density difference (Δρ), which is defined as the difference between the heterostructure electron density ($\rho_{\mathrm{Zn}\text{:}\mathrm{ZnO}}$) and the individual phases ($\rho_{\mathrm{Zn}}$ and $\rho_{\mathrm{ZnO}}$):

(1) $$\Delta {\rho =\rho }_{\mathrm{Zn}\text{:}\mathrm{ZnO}}-{(\rho }_{\mathrm{Zn}}{+\rho }_{\mathrm{ZnO}}),$$

**Figure 2:**
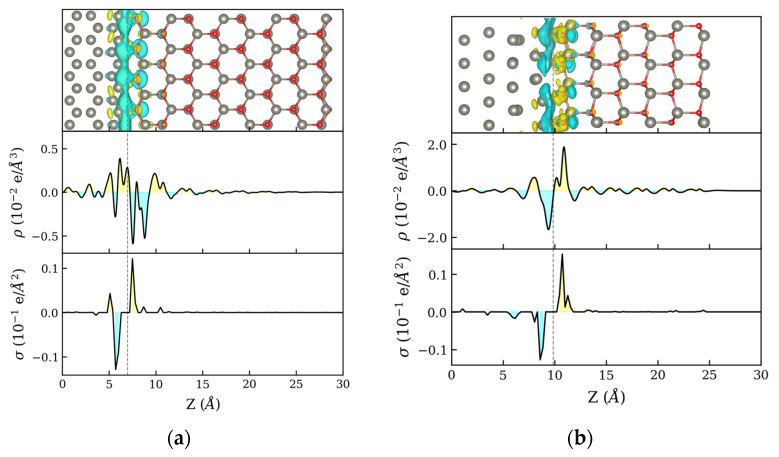
Charge difference along the direction perpendicular to the heterostructure surface (z) for (**a**) NPZnOTH and (**b**) PZnOTH heterostructures.

Figure 2 shows the density difference for NPZnOTH and PZnOTH heterostructures, respectively. Iso-surfaces of charge density differences are shown on the upper plots, where charge accumulation and depletion are represented by the yellow and blue colors, respectively. Zn and O atoms are represented by the gray and red spheres. The center plots refer to the x–y average of the charge density difference in the direction perpendicular to the heterostructure surface (Z). In both systems, charge rearrangement is observed at the Zn:ZnO interface. On the PZnOTH, it is clear that electron transfer occurs from Zn to the ZnO surface showing well-defined regions of charge accumulation (ZnO side) and depletion (Zn side). The NPZnOTH, charge density difference profile is somewhat more complex due to the interaction of Zn and O atoms from ZnO phase with the atoms of metallic Zn. To better understand the global charge balance in the heterostructures, a Bader charge analysis was performed by calculating the charge variation of each individual atom. Figure 2 bottom plots show the planar density profile of the Bader charge difference relative to the Z direction. For both NPZnOTH and PZnOTH, the charge redistribution mainly occurs between the first Zn and ZnO layers, with electron accumulation on O atoms of the oxide phase and depletion on atoms of the Zn phase surface.

**Figure 3:**
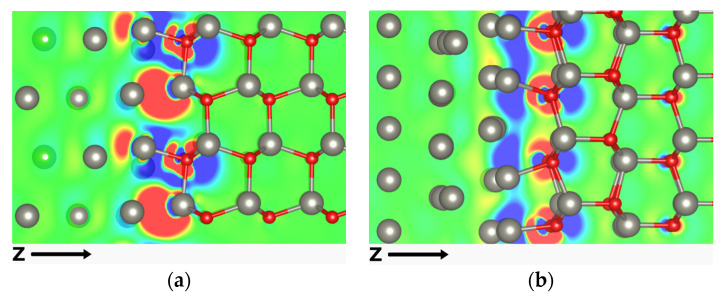
The 2D charge difference plot for NPZnOTH (**a**) and PZnOTH (**b**) heterostructures.

To provide a clearer picture of the bonding at the Zn:ZnO interfaces, a representation of the electron charge density difference on a 2D plane parallel to the Z direction, intercepting Zn and O atoms at the interface, is shown in Figure 3. Electron accumulation and depletion are represented by red and blue regions, respectively. For the O-Zn atom pair formed between the oxide and the metal phases of NPZnOTH (Figure 3a), it is possible to observe electron accumulation on the O atom and depletion around Zn, suggesting the formation of a bond with an ionic character (Zn^2+^ O^2−^ for wurtzite ZnO) [32]. The Zn–Zn interaction at the interface leads to charge depletion of both surfaces in favor of the accumulation in the interfacial space, which can be related to the formation of a metallic bond. For the PZnOTH (Figure 3b), the charge distribution corroborates the formation of a Zn-O bond at the Zn:ZnO interface, with partial ionic character.

**Figure 4:**
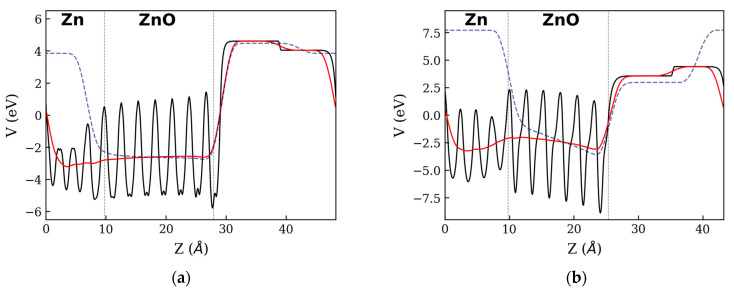
Electrostatic potential (V) for (**a**) NPZnOTH and (**b**) NPZnOTH heterostructures. Vertical dashed lines refer to the limits of each constituting phase of the heterostructures.

Asymmetrical charge distributions between two surfaces of ZnO phase can induce the formation of an electric field inside the oxide layer. Figure 4 shows the x-y planar (solid black lines) and macroscopic (solid red lines) average profile of the electrostatic potential in the direction perpendicular to the heterostructure’s surface (Z). The slope of the macroscopic electrostatic potential profile is indicative of the existence of a potential difference (ΔV) between the two faces of the ZnO phase. The PZnOTH, exhibits a negative slope on the macroscopic potential when going from the Zn:ZnO towards the ZnO:vacuum interface, whereas on the NPZnOTH, the slope is positive. From profiles of the macroscopic potential is possible to estimate the values for ΔV inside the ZnO phase, obtaining ~0.17 eV for NPZnOTH and ~ −0.89 eV for PZnOTH. The macroscopic potential profile of ZnO phase before the contact with the metal is represented as blue dashed lines on Figure 4 It is possible to observe differences on the electrostatic potential before and after the contact with Zn, which translates into changes of ΔV. For NPZnOTH, the ΔV inside the ZnO phase shifts from ~−0.14 to ~0.17 eV and, for PZnOTH, ΔV goes from ~−2.32 to ~−0.89 eV. These changes occur due to the charge accumulation on the ZnO phase (see Figure 2) when a Zn:ZnO interface is formed, lowering the electrostatic potential on the contact side.

### 3.2. O_2_ Adsorption on Zn:ZnO

Adsorption of molecular oxygen was studied on NPZnOTH and PZnOTH heterostructures. The adsorption energy for an oxygen molecule is defined as:

(2) $$E_{\mathrm{ads}}=\left(E_{{\mathrm{Zn}\text{:}\mathrm{ZnO}+O}_{2}}-{\;(E}_{O_{2}}{+E}_{\mathrm{Zn}\text{:}\mathrm{ZnO}})\right)/n$$

where $E_{{\mathrm{Zn}\text{:}\mathrm{ZnO}+O}_{2}}$, $E_{\mathrm{Zn}\text{:}\mathrm{ZnO}}$, and $E_{O_{2}}$ represent the total energies of the Zn:ZnO heterostructure with adsorbed $O_{2}$, the Zn:ZnO clean heterostructure, and the $O_{2}$ monolayer. The adsorption energy per $O_{2}$ molecule is then obtained, dividing the energy difference by the number of oxygen atoms that constitute the adsorbed monolayer (n). A negative adsorption energy indicates favorable adsorption. The calculated E_ads_ also includes the dispersion contributions to the energy. An overview of the relevant aspects concerning ${\;O}_{2}$ adsorption can be found in Table 1, with a separated contribution due to the Van der Waals dispersion forces (ΔVdW).

On the NPZnOTH heterostructure, a low $O_{2}$ adsorption energy was observed, reducing with the increase of the oxide layer number. The adsorption energy also became increasingly dominated by dispersion forces, which was accompanied by a reduction of the charge transfer to the ${\;O}_{2}$ molecule. The bonding distance between the O atoms on the $O_{2}$ molecules was slightly reduced, and its distance to the ZnO surface atoms also increased, reflecting the lower E_ads_. The strength of $O_{2}$ adsorption can be related to the amount of charge that is transferred to the molecule, which is affected by the oxide thickness. On the other hand, the PZnOTH heterostructure showed a strong binding of $O_{2}$ to the ZnO surface, with a small contribution of the dispersion forces. This strong binding can be associated with excess charge on the as-cleaved ZnO(0001) polar surface. This surface presents three-fold coordinated Zn atoms, as opposed to the four-fold coordination that occurs in the bulk. As a result, electron accumulation occurs at the oxide surface, located on the dangling bonds. This charge excess can be removed by different processes, for instance, by the adsorption of atomic species, or via surface rearrangement [33]. Here, the adsorption of $O_{2}$ stabilizes the ZnO surface by removing the existing excess charge. Contrary to what happens on the NPZnOTH, the value of E_ads_ increases with of the number of oxide layers. However, while there is an increase of the adsorption energy, the distance of $O_{2}$ molecule to the oxide surface atoms remains unaffected. The same happens to the O-O distance and to the charge transfer to the $O_{2}$ molecule. This means that the bonding properties between $O_{2}$ and oxide surface ZnO atoms remain unaffected. As such, the observed difference in E_ads_ is not related with the O-Zn bond strength but to the rearrangement at the oxide surface.

The charge redistribution due to the $O_{2}$ adsorption on Zn:ZnO heterostructures was assessed calculating the charge density difference (Δρ) between the heterostructure with adsorbed $O_{2}$ ($\rho_{{\mathrm{Zn}\text{:}\mathrm{ZnO}+O}_{2}}$), and the clean heterostructure ($\rho_{\mathrm{Zn}\text{:}\mathrm{ZnO}}$) plus the isolated $O_{2}$ monolayer ($\rho_{O_{2}}$):

(3) $$\Delta {\rho =\rho }_{{\mathrm{Zn}\text{:}\mathrm{ZnO}+O}_{2}}-{(\rho }_{\mathrm{Zn}\text{:}\mathrm{ZnO}}{+\rho }_{O_{2}}),$$

**Figure 5:**
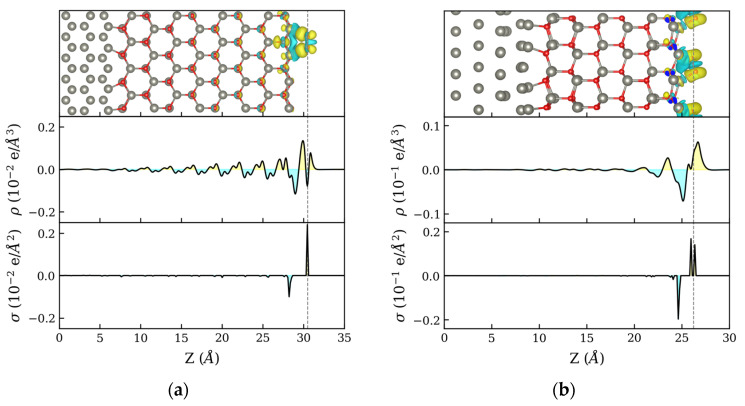
Charge difference, due to oxygen adsorption, on (**a**) NPZnOTH and (**b**) PZnOTH heterostructures.

The profile of Δρ along the direction normal to the interface (Z) is shown in the upper and middle plots of Figure 5. The upper plots show the iso-surfaces for the charge density differences, where charge accumulation and depletion are represented by the yellow and blue colors, respectively. Zn and O atoms are represented by the gray and red spheres. Center plots show the profile of the charge density difference x-y planar average along Z. The analysis of the density profiles of both heterostructures reveals that charge rearrangement occurs on the whole extent of the oxide layer, with very little effect on the metal phase. This suggests that the charge was transferred to the adsorbed $O_{2}$ molecules occur exclusively from the ZnO semiconductor phase with no evidence of other charge transfer mechanisms involving the metal phase, such as electron tunneling. The analysis of the planar density of the Bader charge differences profile along Z (Figure 5, bottom plots) shows, on both NPZnOTH and PZnOTH heterostructures, the formation of charge depletion and charge accumulation regions near the surface. The pronounced depletion region indicates that charge transfer occurs from the heterostructure surface to the adsorbed oxygen.

**Figure 6:**
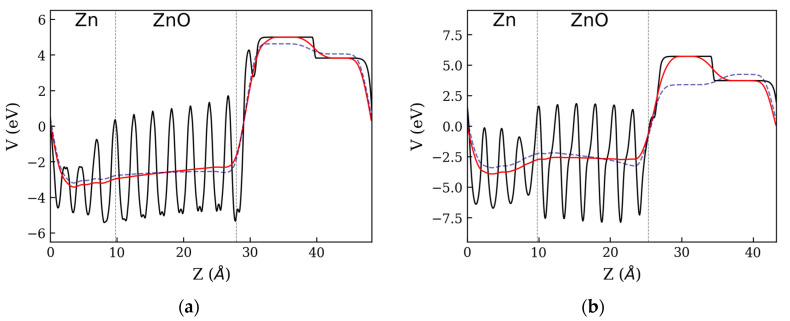
Electrostatic potential (V) for (**a**) NPZnOTH and (**b**) PZnOTH heterostructures.

Figure 6 shows the profile of the electrostatic potential in the direction normal to the NPZnOTH and PZnOTH heterostructures surface, considering $O_{2}$ adsorption. The black lines represent the x-y average of the electrostatic potential (V) for heterostructures with adsorbed oxygen. The solid red lines and dashed blue lines correspond to the macroscopic average after and before $O_{2}$ adsorption. The macroscopic average profile inside the ZnO phase on the NPZnOTH heterostructure shows a positive slope when going from the Zn:ZnO to the ${\mathrm{ZnO}\text{:}O}_{2}$ interface. The ΔV between the two interfaces of the ZnO phase is estimated to be ~0.66 eV, which is higher than the value ΔV before $O_{2}$ adsorption (~0.17 eV). On PZnOTH heterostructure, where a change of ΔV form ~−0.89 to ~−0.2 eV inside the ZnO phase occurs, when $O_{2}$ is adsorbed. The variations observed for ΔV on both heterostructures are a result of the charge depletion from the oxide layer surface, promoted by the $O_{2}$ adsorption, which raises the electrostatic potential inside the ZnO near the heterostructure’s surface.

## 4. Conclusions

Zn:ZnO heterostructures are interesting systems due to their potential range of applications. It is crucial to understand the mechanisms underlying the oxidation process that occurs on this type of heterostructures to help design new devices and applications. Oxidation of Zn under ambient conditions is still a matter of debate, often relying on continuum approach models that don’t consider the atomic configuration or the electronic structure. This work focused on the study of the adsorption of $O_{2}$ molecules on two different surfaces of a Zn:ZnO heterostructure. The adsorption of oxygen on the surface is promoted by charge transfer, resulting in chemical bonds forming. On the NPZnOH, the charge transfer is promoted by electron doping of ZnO due to the formation of the Zn:ZnO interface. On the PZnOH, the charge transfer is promoted by the excess charge located on the ZnO polar surface. In both processes, the charge is removed from the oxide surface and accumulated on the adsorbed $O_{2}$, causing the creation of an electron depletion region. This effect is opposed to the formation at the Zn:ZnO interface, which creates an electron accumulation region on the ZnO side. The difference of electrostatic potential inside the oxide phase is then influenced both by the formation of the Zn:ZnO interface and $O_{2}$ adsorption.

## Figures and Tables

**Table 1:** Properties of $O_{2}$ molecule adsorption on Zn:ZnO heterostructures.

System	ZnO nº Layers	E_ads_ (eV)	ΔVdW (eV)	Q (e^−^)	r(O-O) (Å)	r(Zn1-O1) (Å)	r(Zn2-O2) (Å)
Zn(010):ZnO($10\bar{1}0$) (NPZnOTH)	4	−0.315	−0.138	0.318	1.278	2.264	2.268
	8	−0.213	−0.145	0.165	1.253	2.489	2.491
Zn(001):ZnO(0001) (PZnOTH)	4	−2.432	−0.136	1.133	1.474	1.931	2.041
	6	−2.556	−0.157	1.134	1.473	1.935	2.044

ΔVdW = dispersion energy; Q(e-) = charge transferred to $O_{2}$ molecule (from Bader analysis); r(O-O) = bond length of $O_{2}$ molecule; r (Zn1-O1) and r (Zn2-O2), distance from $O_{2}$ atoms to ZnO surface atoms.

## Data Availability

Data supporting the results presented in this paper will be provided by the corresponding author upon reasonable request.

## Supplementary Materials

The following supporting information can be downloaded at: https://www.mdpi.com/article/10.3390/ma16031275/s1, Figure S1: Zn and ZnO unit cells, Figure S2: Band structure and total DOS for (**a**) ZnO and (**b**) Zn. Table S1: Optimized lattice parameters for Zn and ZnO structures.

## References

[1] Khan W.S., Cao C., Chen Z., Nabi G. (2010). Synthesis, Growth Mechanism, Photoluminescence and Field Emission Properties of Metal–Semiconductor Zn–ZnO Core–Shell Microcactuses. Mater. Chem. Phys..

[2] Moezzi A., McDonagh A.M., Cortie M.B. (2012). Zinc Oxide Particles: Synthesis, Properties and Applications. Chem. Eng. J..

[3] Wang Z., Wang F., Lu Y., Xu M., Li Q. (2016). Induction of Zinc Particles on the Morphology and Photoluminescent Property of Globular Zn/ZnO Core/Shell Nanorod Heterojunction Array Architectures. J. Exp. Nanosci..

[4] Lupan O., Chow L., Chai G., Heinrich H. (2008). Fabrication and Characterization of Zn-ZnO Core-Shell Microspheres from Nanorods. Chem. Phys. Lett..

[5] Khan W.S., Cao C., Nabi G., Yao R., Bhatti S.H. (2010). Catalyst-Free Combined Synthesis of Zn/ZnO Core/Shell Hollow Microspheres and Metallic Zn Microparticles by Thermal Evaporation and Condensation Route. J. Alloys Compd..

[6] Zhang X.Y., Dai J.Y., Lam C.H., Wang H.T., Webley P.A., Li Q., Ong H.C. (2007). Zinc/ZnO Core-Shell Hexagonal Nanodisk Dendrites and Their Photoluminescence. Acta Mater..

[7] Hu J.Q., Li Q., Meng X.M., Lee C.S., Lee S.T. (2003). Thermal Reduction Route to the Fabrication of Coaxial Zn/ZnO Nanocables and ZnO Nanotubes. Chem. Mater..

[8] Gao P.X., Lao C.S., Ding Y., Wang Z.L. (2006). Metal/Semiconductor Core/Shell Nanodisks and Nanotubes. Adv. Funct. Mater..

[9] Calderon V.S., Gomes B., Ferreira P.J., Carvalho S. (2017). Zinc Nanostructures for Oxygen Scavenging. Nanoscale.

[10] Landolt D. (2007). Corrosion and Surface Chemistry of Metals. Mater. Today.

[11] Zhdanov V.P., Kasemo B. (2008). Cabrera–Mott Kinetics of Oxidation of Nm-Sized Metal Particles. Chem. Phys. Lett..

[12] Ermoline A., Dreizin E.L. (2011). Equations for the Cabrera–Mott Kinetics of Oxidation for Spherical Nanoparticles. Chem. Phys. Lett..

[13] Zhdanov V.P., Kasemo B. (2012). Cabrera-Mott Kinetics of Oxidation of Metal Nanowires. Appl. Phys. Lett..

[14] Mahapatra A.K., Bhatta U.M., Som T. (2012). Oxidation Mechanism in Metal Nanoclusters: Zn Nanoclusters to ZnO Hollow Nanoclusters. J. Phys. D Appl. Phys..

[15] Mora-Fonz D., Lazauskas T., Farrow M.R., Catlow C.R.A., Woodley S.M., Sokol A.A. (2017). Why Are Polar Surfaces of ZnO Stable?. Chem. Mater..

[16] Ding Y., Kong X.Y., Wang Z.L. (2004). Interface and Defect Structures of Zn-ZnO Core-Shell Heteronanobelts. J. Appl. Phys..

[17] Sołtys J., Piechota J., Łopuszyński M., Krukowski S. (2013). Density Functional Theory (DFT) Study of Zn, O2 and O Adsorption on Polar ZnO(0001) and ZnO (0001) Surfaces. J. Cryst. Growth.

[18] Ma D., Wang Z., Cui H., Zeng J., He C., Lu Z. (2016). First-Principles Study of O2 Adsorption on Al-Doped ZnO(1010) Surface. Sens. Actuators B Chem..

[19] Perdew J.P., Burke K., Ernzerhof M. (1996). Generalized Gradient Approximation Made Simple. Phys. Rev. Lett..

[20] Kresse G., Furthmüller J. (1996). Efficient Iterative Schemes for Ab Initio Total-Energy Calculations Using a Plane-Wave Basis Set. Phys. Rev. B Condens. Matter Mater. Phys..

[21] Blöchl P.E. (1994). Projector Augmented-Wave Method. Phys. Rev. B.

[22] Grimme S., Ehrlich S., Goerigk L. (2011). Effect of the Damping Function in Dispersion Corrected Density Functional Theory. J. Comput. Chem..

[23] Bashyal K., Pyles C.K., Afroosheh S., Lamichhane A., Zayak A.T. (2018). Empirical Optimization of DFT + U and HSE for the Band Structure of ZnO. J. Phys. Condens. Matter.

[24] Hinuma Y., Grüneis A., Kresse G., Oba F. (2014). Band Alignment of Semiconductors from Density-Functional Theory and Many-Body Perturbation Theory. Phys. Rev. B.

[25] Ma X., Wu Y., Lv Y., Zhu Y. (2013). Correlation Effects on Lattice Relaxation and Electronic Structure of ZnO within the GGA+ U Formalism. J. Phys. Chem. C.

[26] Hinuma Y., Oba F., Kumagai Y., Tanaka I. (2013). Band Offsets of CuInSe2/CdS and CuInSe2/ZnS (110) Interfaces: A Hybrid Density Functional Theory Study. Phys. Rev. B Condens. Matter Mater. Phys..

[27] Colombo L., Resta R., Baroni S. (1991). Valence-Band Offsets at Strained Si/Ge Interfaces. Phys. Rev. B.

[28] Momma K., Izumi F. (2011). VESTA 3 for Three-Dimensional Visualization of Crystal, Volumetric and Morphology Data. J. Appl. Crystallogr..

[29] Henkelman G., Arnaldsson A., Jónsson H. (2006). A Fast and Robust Algorithm for Bader Decomposition of Charge Density. Comput. Mater. Sci..

[30] D’Amico N.R., Cantele G., Perroni C.A., Ninno D. (2015). Electronic Properties and Schottky Barriers at ZnO–Metal Interfaces from First Principles. J. Phys. Condens. Matter.

[31] Todorova M., Neugebauer J. (2015). Identification of Bulk Oxide Defects in an Electrochemical Environment. Faraday Discuss..

[32] Morkoç H., Özgür Ü. (2009). Zinc Oxide.

[33] Gorai P., Seebauer E.G., Ertekin E. (2016). Mechanism and Energetics of O and O 2 Adsorption on Polar and Non-Polar ZnO Surfaces. J. Chem. Phys..

