# Supplementary material for: Oxygen Adsorption on Polar and Non-Polar Zn:ZnO Heterostructures from First Principles
Source: Materials (Basel). 2023 Feb 2;16(3):1275. doi: 10.3390/ma16031275 (PMC9919104; doi:10.3390/ma16031275)
Supplement: Supplementary file 1 [file materials-16-01275-s001.zip › materials-2161435-supplementary.pdf]

materials

MDPI

Article

Oxygen Adsorption on Polar and Non-Polar Zn:ZnO Heterostructures from First Principles

António Castro [1,2], Sebastian Calderon [3,†] and Luís Marques [1,2,*]

1 Center of Physics of Minho and Porto Universities, University of Minho, Campus de Gualtar, 4710-057 Braga, Portugal
2 Laboratory of Physics for Materials and Emergent Technologies, LapMET, University of Minho, 4710-057 Braga, Portugal
3 INL, International Iberian Nanotechnology Laboratory, Av. Mestre José Veiga, 4715-330 Braga, Portugal
* Correspondence: lsam@fisica.uminho.pt
† Current address: Department of Materials Science and Engineering, Carnegie Mellon University, Pittsburgh, PA 15213, USA

DFT Calculations hcp Zn and Wurtzite ZnO

Calculations with spin polarization were performed within the density functional theory (DFT). The exchange correlation potentials were treated using the Perdew-Burke-Ernzerhof (PBE) parameterization within the general gradient approximation (GGA), as implemented in the Vienna ab initio simulation package (VASP), with the frozen-core projector-augmented-wave (PAW) pseudopotentials. A plane-wave basis set was used with an energy cut-off of 520 eV and Gaussian smearing was used with $\sigma = 0.02$ eV. Dispersion forces were taken into account under the DFT-D3 method of Grimme with Becke-Johnson damping. Zn an ZnO unit cells were optimized with a conjugate gradient method, allowing. Atom positions and cell dimension to relax until atomic forces were less than 0.001 eV/$\AA$. Brillouin integration was performed using a Monkhorst-Pack mesh with k-point sampling using a 19 × 19 × 11 mesh for hcp Zinc and 15 × 5 × 9 for wurtzite ZnO.

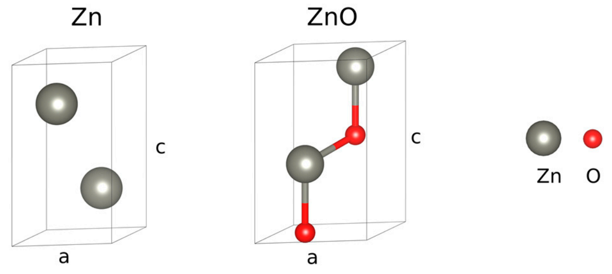

Figure S1. Zn and ZnO unit cells.

Table S1. Optimized lattice parameters for Zn and ZnO structures.

System	Parameter	GGA-PBE + D	Experimental
Zn	a ($\AA$)	2.627	2.665
	c ($\AA$)	4.774	4.947
ZnO	a ($\AA$)	3.262	3.249
	c ($\AA$)	5.269	5.206

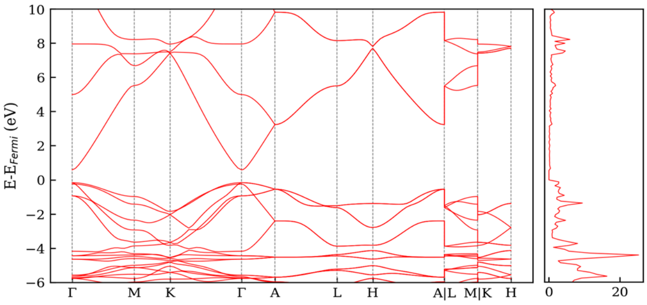

(**a**)

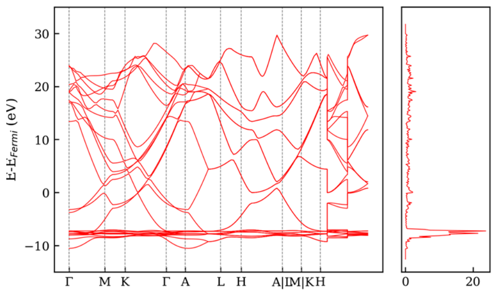

(**b**)

Figure S2. Band structure and total DOS for (**a**) ZnO and (**b**) Zn.
